# Influence of Spray-dried Hydroxyapatite-5-Fluorouracil Granules on Cell Lines Derived from Tissues of Mesenchymal Origin

**DOI:** 10.3390/molecules13112729

**Published:** 2008-11-01

**Authors:** Tim Scharnweber, Catarina Santos, Ralf-Peter Franke, Maria Margarida Almeida, Maria Elisabete V. Costa

**Affiliations:** 1 Institute for Biological Interfaces; Forschungszentrum Karlsruhe GmbH, 76344 Eggenstein-Leopoldshafen Karlsruhe, Germany; 2 Department of Ceramic and Glass Engineering, CICECO, University of Aveiro, 3810-193 Aveiro, Portugal; E-mails: cfsantos@ua.pt (C. S.), margarida@ua.pt (M-M. A.), elisabete.costa@ua.pt (M-V. C.); 3 Department of Biomaterials, University of Ulm, 89081 Ulm, Germany; E-mail: ralf-peter.franke@uni-ulm.de (R-P. F.)

**Keywords:** Hydroxyapatite, Spray dried particles, 5-Fluorouracil, in Vitro cytotoxicity, Drug delivery system

## Abstract

In our previous work we described the preparation and characterization of spray dried hydroxyapatite micro granules loaded with 5-fluorouracil (5-FU). These loaded particles are used as a model drug delivery system (DDS). In this study we examined the *in vitro* response of two cell lines derived from different tissues to 5-FU loaded granules (LG). Both cell lines, either L929 cells of a mouse fibroblast lineage or cells originating from a rat osteosarcoma (ROS 17/2.8) showed a dose dependent decrease in cell proliferation in response to 5-FU-, either dissolved in the culture medium or loaded onto particles. The response of the two cell lines to loaded and nonloaded particles was different. The effect of LG and of a corresponding concentration of free 5-FU was practically the same for the ROS 17/2.8 cells indicating that ROS 17/2.8 cells were not affected by the carrier material. In contrast, L929 cells showed a slight decrease in cell proliferation also in the presence of granules not loaded with 5-FU. This is thought to be attributed to the inhibition of mitogenesis by phosphocitrates, already demonstrated in fibroblasts. In summary, we found that the loaded 5-FU kept its effectivity after the spray drying process and that the response towards the granules varied with cell type. This is the first step towards a tissue specific DDS.

## Introduction

The chemical similarity of hydroxyapatite [HAP, Ca_10_(PO_4_)_6_(OH)_2_] to the calcium phosphate phases in bone and its excellent biocompatibility make it an attractive biomaterial for medical applications. Of great importance is the ability of HAP to adsorb and release various molecules of biological and medical interest [1]. Besides the possibility to load apatite implant materials with pharmaceuticals for a constant and controlled drug release, there is also a potential to use small HAP particles as carriers. Such particles could be administered directly into the affected tissue. The advantages of such particulate DDS lie in an increased bioavailability and a predictable therapeutic response, as well as a controlled and prolonged release time only at the place where the drug is needed. This allows a reduction of the total amount of drug applied which in return can reduce the incidence of adverse reactions, which may be severe as is the case for many anti cancer drugs.

Interestingly, it was reported that even unloaded HAP nanoparticles could have an inhibitory effect on various tumor cells [2,3,4]. To affect a certain cell type or to increase their effect, nanoparticles can also be loaded with drugs. One example is the use of phosphonates which have a high affinity towards HAP making a surface modification of the particles unnecessary [5].

For the production of HAP microgranules a number of methods have been described. A common approach to produce porous granules is based on the effect of liquid immiscibility, where a suspension of hydroxyapatite in an aqueous medium is dispersed in oil or paraffin [6, 7]. Other production techniques include dripping [8], drip casting [9] or spray drying [10, 11]. Spray drying is a fast and straightforward process that allows the production of granules [12, 13] loaded with drugs in a single step. The preparation of porous HAP granules loaded with the drug 5-fluorouracil (5-FU) has already been reported [14]. The methodology allows the preparation of homogeneous microgranules with a uniform size, where the molecular integrity of the drug as well as the bulk properties of the ceramic were maintained, indicating that the 5-FU did not induce any modifications in the structure of the hydroxyapatite. 5-FU is an antineoplastic agent with a relatively short (10-20 min) plasma half-life and commonly used in the therapy of different solid tumor types [15]. 

The objective of the present work was the investigation of the *in vitro* response of different cell lines to the DDS. As different tissues may react differently to a pharmaceutical agent two cell lines, one of the osteoblastic lineage and the other of a fibroblastic lineage have been used. The tests were performed with three types of granules: unloaded granules (UG) used as controls and 5-FU loaded granules produced at spray drying temperatures of 80°C (LG80) and 120°C (LG120), respectively.

## Results and Discussion

**Figure 1:**
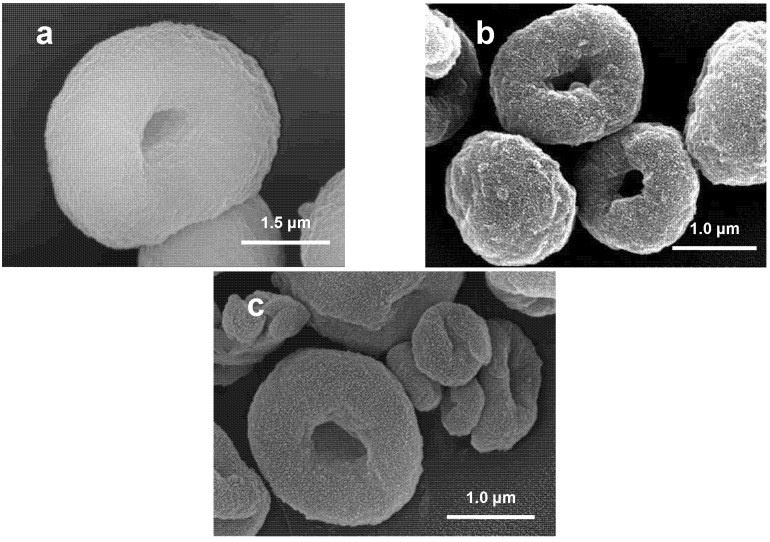
SEM images of spray dried granules representative of unloaded granules produced at 80°C (a), loaded granules produced at 80°C (b), and at 120°C (c), respectively.

**Figure 2:**
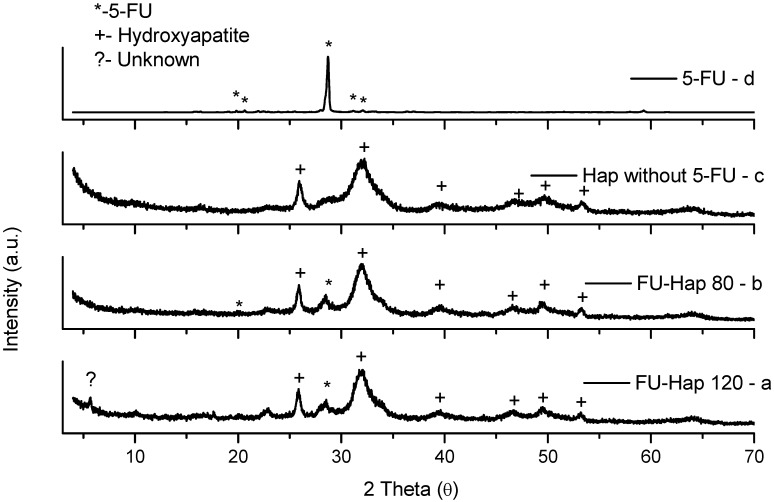
X-ray diffraction patterns of spray dried Hap-FU granules at 120ºC (a); 80ºC (b); unloaded granules (UG) (c); pure 5-FU (d).

According to preceding studies [11] all three granule types have a regular donut shape (Figure 1). In the case of LG120 minor amounts of scattered rod like particles were also found (Figure 1c). The X-ray diffraction patterns of the spray-dried powders (Figure 2) in most cases displayed the presence of crystalline HAP and 5-FU phases and no detectable decomposition products [14], except LG120 which revealed traces of an unknown phase (Figure 2a).

Previous results [14] also showed that the release of the drug from LG80 in phosphate buffered saline at 37°C was fast. Furthermore, the amount of 5-FU released corresponded approximately to the total load of the granules. This led to the assumption that 5-FU existed as a solid phase in accessible regions of the granule.

For the study of the *in vitro* effect of 5-FU loaded granules with different concentrations of UG, LG80, LG120, and the drug 5-FU alone were added either to a cell culture of fibroblastic L929 cells or to ROS 17/2.8 cells of the osteoblastic lineage. These two cell lines used in this study could be shown to be applicable for this test. Since different body tissues may react in very different ways towards external stimuli and drugs, we have chosen cells originating from two relevant body tissues (connective tissue and bone). As a derivative of the nucleobase uracil, 5-FU is incorporated into DNA and RNA, finally resulting in cell death by apoptosis. Thus proliferating cells are most susceptible to the cytotoxicity of the drug. The decrease in cell proliferation has been assessed in cells growing for a defined period of time, then comparing the yield of control cells with that of cells in contact with granules and / or drugs (free 5-FU, LG80, LG120, UG). So our approach to evaluate the cytotoxicity was an indirect one as we drew our conclusions from the change in cell growth only. Nevertheless our observations concerning the pathway of cell death were according to the expectations. Cells in contact with high concentrations of 5-FU or 5-FU loaded granules showed fragmentation of the nucleus as it is typical for apoptosis. 

**Figure 3:**
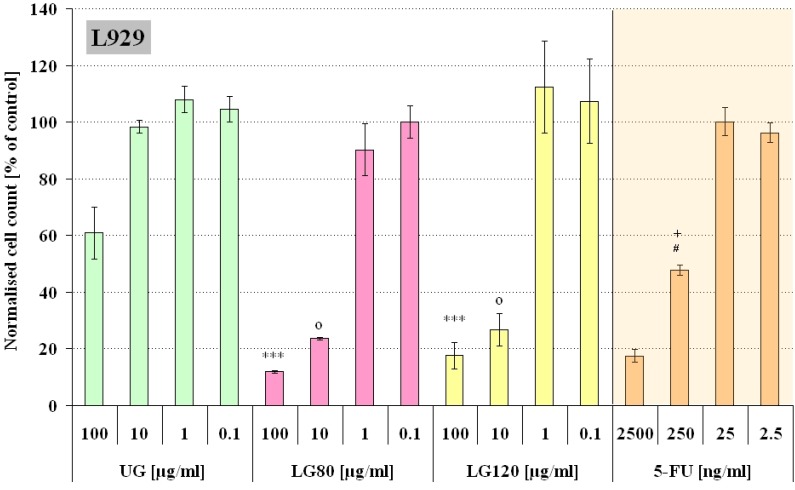
Number of L929 cells incubated for 48h with either unloaded granules (UG), granules containing 5-FU produced at 80°C (LG80) and 120°C (LG120) or free 5-FU of corresponding concentrations. The cell count is expressed as percentage of the control (n=12).

**Figure 4:**
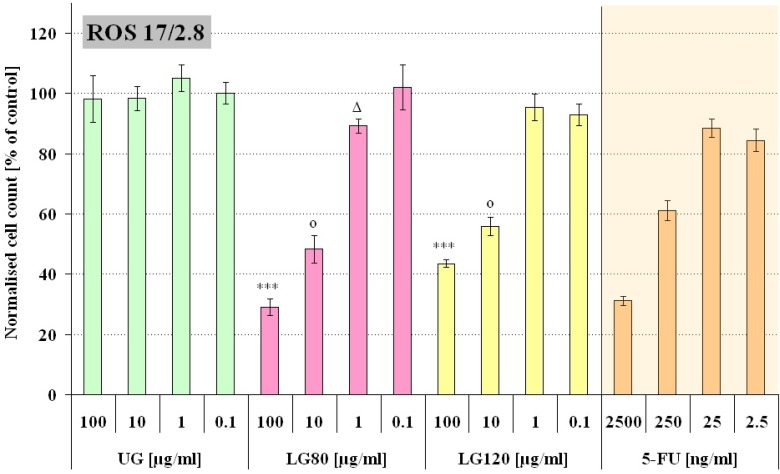
Number of ROS 17/2.8 cells incubated for 72h with either unloaded granules (UG), granules containing 5-FU produced at 80°C (LG80) and 120°C (LG120) or free 5-FU of corresponding concentrations. The cell count is expressed as percentage of the control (n=12).

Figure 3 and Figure 4 summarize the obtained results. The cells were incubated with four different concentrations of granules in the cell culture medium (100, 10, 1, and 0.1 µg/mL). The concentrations of the free 5-FU was chosen to match the 5-FU concentrations that could be released from the corresponding loaded granules in case of complete release (1g LG contained approximately 25 mg 5-FU). Based on preceding studies concerning the drug release properties of the granules it is expected that nearly the complete amount of loaded 5-FU was released during the time of incubation [14].The statistical significance of the effect of loaded and unloaded granules was tested with a parametric ANOVA test. For both cell lines there was a significant difference between the two highest concentrations of UG compared to LG80 and LG120, respectively (p<0.001, see Figure 3 and Figure 4). From this we can conclude that a concentration of 10 µg/mL or higher of loaded granules has a different impact on the cells than granules without a drug load. A pairwise comparison of the corresponding 5-FU concentrations (LG80, LG120 and free 5-FU) revealed only one significant difference between the data sets in L929 cells (LG80/LG120 10 µg/mL vs. 250 ng/mL 5-FU, p<0.01) and none in ROS cells. This demonstrates that the observed adverse effect on the final cell number can most likely be attributed to the cytotoxicity of the released 5-FU. 

***: UG 100 µg/mL vs. LG80 100 µg/mL, LG120 100 µg/mL p<0.001

o: UG 10 µg/mL vs. LG80 10 µg/mL, LG120 10 µg/mL p<0.001

#: LG80 10 µg/mL vs. 250 ng/mL 5-FU p<0.01; +: LG120 10 µg/mL vs. 250 ng/mL 5-FU p<0.05

**Figure 5:**
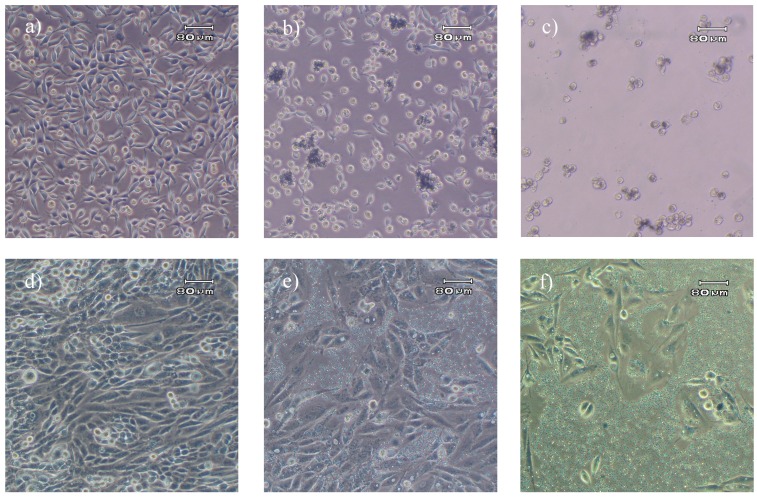
Comparison of L929 fibroblast cells and ROS 17/2.8, 72 h and 96 h after seeding, respectively. a) L929 control without granules. b) L929 incubated for 48 h in the presence of 100 µg/mL granules without 5-FU (UG). c) L929 incubated for 48 h in the presence of 100 µg/mL granules loaded with 5-FU (LG80). d) ROS 17/2.8 control without granules. e) ROS 17/2.8 cells cultivated for 72 h in the presence of 100 µg/mL UG. f) ROS 17/2.8 cells cultivated for 72 h in the presence of 100 µg/mL LG80

It was noticeable that unloaded granules had a significant inhibitory influence on cell growth of L929 cells (green columns, Figure 3), but not on ROS cells. This may be caused by the different behaviour of the two cell lines towards the granules as particulate matter. L929 cells after three days of culture in UG-containing medium were clustered around particle aggregates, but the majority of particles seemed to be depleted from the medium (Figure 5b). Based on the knowledge that fibroblasts can be able to phagocitose [16] and on the fact that for L929 cells specifically the cellular uptake of particulate matter has been well documented [17,18] it can be concluded that a great amount of loaded as well as unloaded granules, were internalized by L929 cells via phagocytosis. ROS cells on the other hand were not shown to internalise the particles: Free distributed particles were visible in the culture even after 72h incubation (Figure 5e+f).

***: UG 100 µg/mL vs. LG80 100 µg/mL, LG120 100 µg/mL p<0.001

o: UG 10 µg/mL vs. LG80 10 µg/mL, LG120 10 µg/mL p<0.001

∆: UG 1 µg/mL vs. LG80 1 µg/mL p<0.01

In L929 cultures the presence of 100 µg/mL UG led to a significant change in cell morphology. The majority of the cells developed a round shape (Figure 5b) and only a small number maintained the spindle shaped morphology that is typical of fibroblasts and L929 cells (Figure 5a). It has been reported in literature that small calcium phosphate particles with a mean size below 4 µm had a toxic effect on primary rat fibroblasts [19]. However, the authors observed this effect at high particle concentrations which were more than ten times higher than the maximal concentration used in this study. And they attributed this adverse effect of the phagocytosed particles on cell growth rather to physical effects than to chemical ones. In contrast, lower concentrations of calcium phosphate comparable to those used in our experiments were reported to increase proliferation [17, 20]. Now, a somewhat similar trend was apparent in our results with a slight increase in proliferation at low concentrations of UG in L929 cultures and an intermediate decrease at the maximum concentration of UG used here in L929 cultures. The proliferation increase at low UG concentrations could relate to the same phenomena already described [17, 20] for calcium phosphate particles. Our granules, however, were produced from nanoparticles which contained citrate. Citrate is used as an inhibitor of hydroxyapatite crystallization during the production of HAP nanoparticles.

The resulting phosphocitrates can act as an antagonist to the mitogenic activity of pure hydroxyapatite and might level or even decrease cell proliferation [21, 22]. At the concentrations of UG here used in L929 cultures which were rather low in comparison to those used in [19], the results from our examination suggest that the increasing amount of phosphocitrates coinciding with an increase in particle concentration caused the decrease in numbers of L929 cells.

It is further anticipated that both effects, the negative effect of phagocytosed phosphocitrates and the toxicity of 5-FU summed up in L929 cells and led to a stronger reduction of proliferation and cell numbers in L929 cells compared to ROS 17/2.8 cells after incubation with 5-FU loaded granules at higher concentrations.

Interestingly, the ROS 17/2.8 cells were not affected by the unloaded granules. Cells in contact with 100 µg/mL UG (Figure 5e) displayed their characteristic morphology and no phagocytotic activity could be observed. The cells observed seemed to have moved aside the greatest part of the particles while they proliferated. This may explain why ROS 17/2.8 cells were not affected by the phosphocitrate. ROS 17/2.8 cells in contact with non-loaded granules proliferated at the same rate as cells in particle free medium (Figure 4). 

From XRD results it was detected that the crystallinities of the granules produced at 80 and 120°C respectively were slightly different, so, it was interesting to examine if this difference had an impact on our *in vitro* system. Indeed, there were differences in the response of L929 and ROS cells to LG80 and LG120. L929 cells were clearly more sensitive to both, the granules (Figure 3: UG, LG80, LG120) and the 5-FU (Figure 3 and Figure 4: 5-FU). From this perspective, the increased proliferation in L929 cells exposed to low doses of LG120 (Figure 3) does not seem to be due to the presence of low dose 5-FU, because 5-FU in low doses alone did not elicit similar effects. It can be speculated therefore, that this effect might be due to changes in structure and crystallinity of the granules after the heat treatment at elevated temperature. The effective principle, however, needs to be established in future examinations.

Our results show that the spray drying process did not affect the effectivity of 5-FU. Furthermore, HAP microgranules loaded with 5-FU efficiently reduced the proliferation of cancer cells *in vitro*. For cell types like fibroblasts, that are able to endocytose, the adverse effect on cell proliferation was more pronounced probably due to the additional inhibition of mitosis caused by the internalization of phosphocitrate. By adjusting the release rate so that the predominant part of the chemotherapeutic drug is released at the location of the tumor, the systemical effects would be minimized and the efficiency enhanced. So, further work will focus on the tailoring of the drug release rate. A problem might arise if granules are not internalized by the tumor cells. This could result in free circulation of granules through the body which might impair healthy tissue as even granules that have released their cargo might disturb cell proliferation as was shown for L929 cells. Studies are now in progress aiming to clarify the reasons underlying the adverse effect on cell proliferation of the unloaded granules. Such studies are expected to improve the granules production, so that fully biocompatible materials (granules) can be obtained. The differing responses of the cell lines highlight the fact that for successful design of a DDS one has to consider the special characteristics of the diseased tissues as well as their environment to optimize the effect. It can already be concluded that HAP microgranules are interesting candidates for DDS. As the production process of loaded microgranules is fast, easy and cost effective, further studies are very interesting from the economical point of view.

## Experimental

### Preparation and characterization of 5-FU-loaded particles

The loaded particles were produced as described elsewhere [14]. In brief, an aqueous solution of 5-FU (Sigma, Portugal) with a concentration of 250 mg/L was used for dispersing nanosized HAP particles (1 wt.%), that were obtained by a precipitation process [11, 23]. This corresponds to a theoretical 5-FU load of 25 mg/g of granules. This suspension was used to feed a laboratory spray dryer (Büchi B-191). The resulting spray dried powders were collected from the cyclone and used for in vitro experiments. The granules were produced at a spray drying temperature of 80°C and 120ºC either loaded with 5-FU (LG80) or unloaded (control, UG). The powders were characterized with respect to morphology (scanning electron microscopy), crystallinity (X-ray diffraction), and the presence of 5FU was optically verified by fluorescence microscopy [14].

### In vitro cytotoxicity testing in L929 fibroblasts and osteosarcoma ROS cells

The mouse fibroblast cell line L929 (ATCC Catalog No. CCL-1) was grown in Eagle’s Minimal Essential Medium (ATCC, Catalog No. 30-2003), 1% penicillin/streptomycin (PAA) and 10% horse serum (ATCC Catalog No.30-2040). The cells were split twice a week with 0.2% trypsin solution containing 0.6% EDTA (Invitrogen) at about 90% confluency at a ratio of 1:4.

The rat derived osteosarcoma cell line ROS 17/2.8 was grown in Dulbecco's Modified Eagle Medium, supplemented with 1 mM sodium pyruvate, 1% penicillin/streptomycin and 10% fetal calf serum, all obtained from PAA. The cells were split at confluency with a 0.05% trypsin solution containing 0.15% EDTA once a week at a ratio of about 1:20 to 1:40. All cells were incubated at 37°C in a humid atmosphere with 5% CO_2_.

Confluent cell populations of L929 and ROS 17/2.8 were harvested with the same trypsin/EDTA solutions as for splitting and counted in a CASY^®^ cell counter (Schärfe System GmbH). The cell suspension was diluted with culture medium to 10^5^ cells per milliliter and plated into a 24-well culture plate (1mL/well). Cells were allowed to spread and adapt to the new environment for 24h. After controlling their uniform distribution throughout the plates by light microscopy, the medium was removed and replaced with fresh culture medium (controls) or with culture medium supplemented with different concentrations of the samples. The samples containing suspended microgranules (LG80 and LG120) with or without 5-FU (UG) were prepared in four concentrations: 100, 10, 1 and 0.1 µg/mL. 5‑FU (Hexal) was applied in concentrations corresponding to the maximum amount that could be released from the particles used in the assay. In addition, two concentrations above and below this range were tested.

As the proliferation rates of the cell lines utilized differed slightly, the experiments were stopped at a point where the controls were still in the phase of exponential growth. This was after 48h in the case of L929 and 72h for ROS 17/2.8. Cells were harvested from the wells with trypsin/EDTA and counted. All samples were tested in quadruplicate. On every plate there was a row of controls to check for the general cell performance. All cell counts were normalized to the controls. The experiments were repeated three times.
